# Limited Sampling Strategy for Determination of Ibrutinib Plasma Exposure: Joint Analyses with Metabolite Data

**DOI:** 10.3390/ph14020162

**Published:** 2021-02-18

**Authors:** Félicien Le Louedec, Fanny Gallais, Fabienne Thomas, Mélanie White-Koning, Ben Allal, Caroline Protin, Loïc Ysebaert, Étienne Chatelut, Florent Puisset

**Affiliations:** 1 Department of Pharmacology, Institut Claudius Régaud, Institut Universitaire du Cancer de Toulouse—Oncopole, 31059 Toulouse, France; thomas.fabienne@iuct-oncopole.fr (F.T.); allal.ben@iuct-oncopole.fr (B.A.); chatelut.etienne@iuct-oncopole.fr (É.C.); 2 Cancer Research Center of Toulouse, Inserm UMR1037, 31037 Toulouse, France; fanny.gallais@univ-tlse3.fr (F.G.); melanie.white-koning@univ-tlse3.fr (M.W.-K.); ysebaert.loic@iuct-oncopole.fr (L.Y.); puisset.florent@iuct-oncopole.fr (F.P.); 3 Université Paul Sabatier Toulouse III, 31062 Toulouse, France; 4 Department of Hematology, Institut Universitaire du Cancer de Toulouse—Oncopole, 31059 Toulouse, France; protin.caroline@iuct-oncopole.fr; 5 Department of Pharmacy, Institut Universitaire du Cancer de Toulouse—Oncopole, 31059 Toulouse, France

**Keywords:** ibrutinib, metabolite, therapeutic drug monitoring, Bayesian analysis, pharmacokinetics

## Abstract

Therapeutic drug monitoring of ibrutinib is based on the area under the curve of concentration vs. time (AUC_IBRU_) instead of trough concentration (C_min,ss_) because of a limited accumulation in plasma. Our objective was to identify a limited sampling strategy (LSS) to estimate AUC_IBRU_ associated with Bayesian estimation. The actual AUC_IBRU_ of 85 patients was determined by the Bayesian analysis of the full pharmacokinetic profile of ibrutinib concentrations (pre-dose T0 and 0.5, 1, 2, 4 and 6 h post-dose) and experimental AUC_IBRU_ were derived considering combinations of one to four sampling times. The T0–1–2–4 design was the most accurate LSS (root-mean-square error RMSE = 11.0%), and three-point strategies removing the 1 h or 2 h points (RMSE = 22.7% and 14.5%, respectively) also showed good accuracy. The correlation between the actual AUC_IBRU_ and C_min,ss_ was poor (*r*^2^ = 0.25). The joint analysis of dihydrodiol-ibrutinib metabolite concentrations did not improve the predictive performance of AUC_IBRU_. These results were confirmed in a prospective validation cohort (*n* = 27 patients). At least three samples, within the pre-dose and 4 h post-dose period, are necessary to estimate ibrutinib exposure accurately.

## 1. Introduction

Tyrosine kinase inhibitors (TKIs) are oral drugs given continuously, for most of them once or twice a day, to treat several solid tumors or hematological malignancies. Therapeutic drug monitoring (TDM) is increasingly being used for some TKIs, mainly depending on how long the drugs have been on the market and in accordance with the growing awareness of the benefit of TDM in the management of TKIs. For imatinib, the first TKI to have obtained approval (in 2001), TDM is now routinely performed since disease response (both in chronic myeloid leukemia and gastro intestinal stromal tumors) is highly correlated with exposure to imatinib and is based on trough concentration at steady-state (C_min,ss_) as a surrogate of drug exposure [1,2]. TDM has been generalized to other drugs used to treat chronic myeloid leukemia, such as dasatinib and nilotinib [3]. For the treatment of solid tumors, exposure–treatment outcome (efficacy and toxicity) relationships have been established, and therapeutic windows have been defined for some TKIs [4,5]. For instance, dose optimization based on measured blood concentration has been implemented in routine practice for TKIs such as pazopanib and sunitinib [6].

Ibrutinib (IMBRUVICA^®^) is an irreversible Bruton tyrosine kinase (BTK) inhibitor indicated as a single-agent or in combination regimens for the treatment of chronic lymphocytic leukemia (CLL) [7], mantle cell lymphoma (MCL) [8] and Waldenström’s macroglobulinaemia (WM) [9]. Ibrutinib is approved at the dose of 420 mg/day or 560 mg/day depending on its indication [10].

Preliminary pharmacokinetic/pharmacodynamics (PK/PD) results give a rationale for developing TDM for ibrutinib. Indeed, we have previously reported that patients who discontinued therapy because of the occurrence of an adverse drug reaction had a 1.5-fold higher ibrutinib exposure than patients who did not discontinue treatment [11]. In addition, our team recently published the results of a PK/PD analysis showing that considering the individual plasma ibrutinib concentrations rather than the dose of ibrutinib better described its effect on the absolute lymphocyte count [12]. The results of a dose de-escalation pilot study in real-life CLL patients revealed that lower doses of ibrutinib could decrease the risk of toxicity without impairing efficacy. This suggests the existence of a PK/PD relationship, with an efficacious exposure threshold to ibrutinib lower than the one obtained with usual doses [13].

To date, no target concentration or AUC has been clearly defined for ibrutinib response or toxicity. In such cases, it is usual to refer to the average concentrations or to the AUC observed in the PK studies performed during drug clinical development [14]. For ibrutinib, the mean (SD) exposure was an AUC_τ,SS_ of 680 (517) ng/mL.h in CLL patients treated with 420 mg q.d. during a phase I/II study, and 953 (705) ng/mL.h in MCL patients treated with 560 mg q.d. in a phase II study [15].

Like most TKIs, ibrutinib is eliminated mainly by a hepatic metabolism catalyzed by CYP3A4. It has a short half-life (T_1/2_ 4 to 13 h), due to a high coefficient of hepatic extraction, which is itself responsible for the weak oral bioavailability of the drug (mean 2.9%) [10]. As a consequence of this short T_1/2_, C_min,ss_ is expected to be a poor surrogate of whole plasma exposure for ibrutinib. In addition, its administration schedule (once a day) is associated with limited accumulation. Thus, the AUC must be determined to estimate exposure to ibrutinib. However, this usually requires a high number of blood samples, which is not convenient in routine clinical practice.

The main metabolite of ibrutinib is dihydrodiol-ibrutinib (DHD) which is fifteen times less active on BTK than the parent drug in vitro [15]. However, DHD is less bound to plasma protein than ibrutinib (on average 91% versus 97%) [15], its concentrations are higher than those of ibrutinib, and are very variable between individuals (as for ibrutinib) [11,16]. Thus, the question of also considering DHD concentrations in PK/PD relationships of ibrutinib remains pending.

There is little point in monitoring C_min,ss_ for ibrutinib and reference AUC values observed during clinical development are available. Hence, our objective was to develop a limited sampling strategy (LSS) to estimate ibrutinib AUC using the Bayesian estimation. Pharmacokinetic data from a previous clinical trial were used to evaluate the best limited sampling strategies (LSS) and data from an additional pilot study were used to prospectively evaluate these LSS. We have also considered the data corresponding to DHD, in order to evaluate the benefit of considering DHD plasma concentrations to improve ibrutinib AUC estimates and to refine potential PK/PD relationships.

## 2. Results

### 2.1. Patients

#### 2.1.1. Development Cohort

Of the 89 patients in the cohort used to develop the PK model, 85 were included in the LSS development analysis. Three were excluded due to missing concentration–time points (*n* = 1), outlier concentrations (*n* = 1) or both (*n* = 1). One additional patient was excluded due to an extreme value of C_min,ss_ (53.5 ng/mL) that could have biased the LSS analysis (Grubbs’s test *p* < 10^−15^). Their characteristics are reported in Table 1. Most patients received a dose of 420 mg for one month (1 patient received 560 mg for one month but received 420 mg the day of PK evaluation). The dataset consisted of 1032 concentrations of ibrutinib (516) and DHD (516). Sixteen of them were lower than the lower limit of quantification (LLOQ), mostly trough ibrutinib concentrations (*n* = 12).

#### 2.1.2. Validation Cohort

The validation data consisted of 324 concentrations of ibrutinib (162) and DHD (162), obtained in 27 patients treated with 140 to 420 mg doses, and sampled with the same scheme as in the development cohort. One patient out of the 28 initially available was excluded because one sample was missing. Six ibrutinib concentrations (five residuals) were lower than the LLOQ. Patients’ characteristics are reported in Table 1.

### 2.2. Actual AUC

Actual ibrutinib AUC was obtained after the Bayesian analysis of the full profile of ibrutinib concentrations alone, and not ibrutinib + DHD concentrations. Indeed, in the development data, the correlation between the observed and predicted ibrutinib concentrations was similar, with *r*^2^ = 0.90 when ibrutinib alone concentrations were considered, vs. *r*^2^ = 0.91 for ibrutinib + DHD. Similarly, actual DHD AUC was obtained from the analysis of the DHD concentrations only and not ibrutinib + DHD, because of an equivalent goodness of fit of the DHD concentrations (*r*^2^ = 0.95 vs. 0.92, respectively).

### 2.3. Correlation between trough Ibrutinib Concentrations and Actual AUC

**Figure 1:**
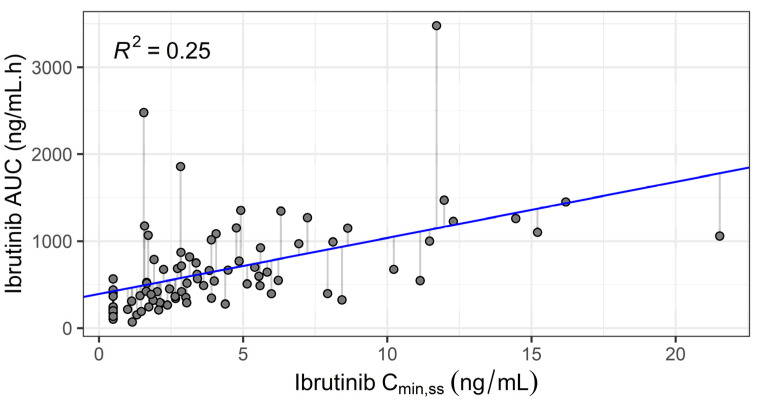
Ibrutinib actual area under the curve (AUC) vs. observed steady-state trough concentration. Solid line: linear regression (*n* = 85 patients).

As expected given its short elimination half-life, the correlation between ibrutinib trough concentrations (C_min,ss_) and actual AUC_IBRU_ was weak (*r*^2^ = 0.25, *n* = 85, see Figure 1). The equation AUC_IBRU_ = 391 + 64.5∙C_min,ss_ (ng/mL) was derived from linear regression.

### 2.4. Limited Sampling Strategies for Determination of Ibrutinib AUC

#### 2.4.1. Development

**Figure 2:**
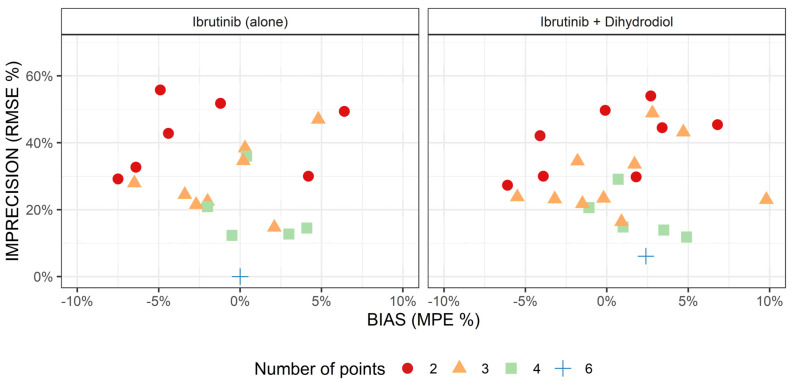
Comparison of the estimation of ibrutinib AUC using ibrutinib concentrations only (**left**) or both ibrutinib and dihydrodiol-ibrutinib (**right**), as a function of the number of points used for estimation (seven values with a bias > 10% are out of bounds). MPE: mean percentage error; RMSE: Root-Mean-Square Error.

Before the determination of the best sampling strategy to predict ibrutinib AUC, we investigated whether considering DHD concentrations would improve the predicting performance of ibrutinib AUC. Figure 2 displays the imprecision and bias of the sampling strategies as a function of the number of sampling points included in the analysis, and whether we analyzed ibrutinib (alone) or both ibrutinib + DHD concentrations. Overall, judging by the graphical inspection, considering metabolite data did not greatly improve the prediction of ibrutinib AUC, for a given number of sampling points. Because DHD concentration measurement is not available in many laboratories, and owing to the parsimony principle, LSS investigation was resumed on analyses including ibrutinib concentrations only.

Table 2 summarizes the performance of the six best sampling strategies, according to the number of points (from 1 to 4), and sorted by increasing RMSE. One- and two-sample strategies were associated with poor performances, with an imprecision of around 50% and 30%, respectively. More specifically, the AUC extrapolated from the single trough concentration of ibrutinib by linear regression was highly biased and imprecise (mean percentage error (MPE) = +42.7%, RMSE = 67.9%). Among the three-sample strategies, T0–1–4 provided the best results with MPE = +2.2% and RMSE = 14.5%. The subsequent strategies were homogeneous in terms of imprecision, with a RMSE of 21% on average. However, differences in MPE were observed: acceptable for T0.5–2–4 and T0–2–4 (MPE = −2.7% and −1.9%, respectively), but unsatisfactory for the T1–4–6, T1–2–4 and T0.5–2–6 strategies (MPE = −7.9%, −6.1% and −9%, respectively). Finally, evaluation of the different four-sample strategies revealed that T0–1–2–4 was superior both in terms of precision (RMSE= 11.0%) and bias (MPE = −0.3%).

#### 2.4.2. Prospective Validation

**Figure 3:**
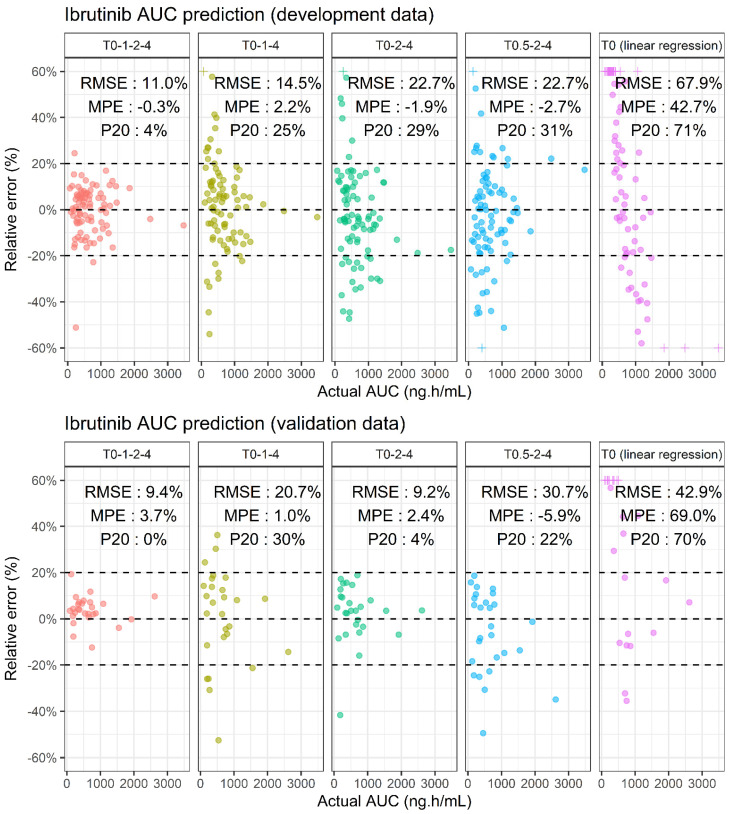
Comparison of the performance of several sampling strategies to predict ibrutinib AUC in the development (**top**) and validation (**bottom**) dataset. Crosses (+) are out of bound values censored to the maximal bound of the y-axis (i.e., +/−60%).

The performance of the T0–1–2–4, T0–1–4, T0–2–4 and T0.5–2–4 sampling strategies were investigated on a validation cohort. These results are presented in Figure 3. The good performance of the four-sample strategy in terms of precision and bias was confirmed, with a RMSE and MPE of 9.4% and +3.7%, respectively. The performance of the three-sample strategies is similar to the results found with the development data, except for the T0.5–2–4 scheme which had higher RMSE and MPE (30.7% and −5.9%, respectively). The results for the T0–2–4 and T0–1–4 strategies remain consistent, except for an inversion in the rank of their respective performances: precision improved to 9.2% for the T0–2–4 LSS but deteriorated from 14.6% to 20.7% for T0–1–4, and the P20 of the T0–2–4 LSS was much lower (4%). Finally, the AUC predicted from the trough concentration and the linear regression was inaccurate (RMSE = 41.0% and MPE = +70.8%).

### 2.5. Anti-BTK AUC

**Figure 4:**
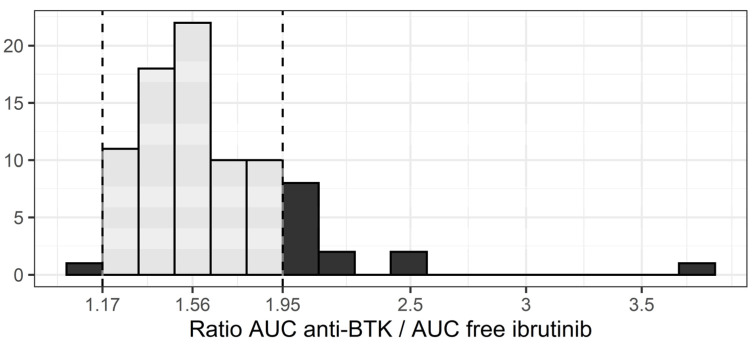
Distribution of the AUC_ANTI-BTK_ / free AUC_IBRU_ ratio (dashed lines: +/−25% interval around the median).

The median ratio between AUC_ANTI-BTK_ and free AUC_IBRU_ was 1.56, with a 22% coefficient of variation (Figure 4). This means that the theoretical contribution of DHD to anti-BTK activity is, on average, equal to half the activity provided by ibrutinib. However, this ratio is not constant for each patient. For instance, one patient with very low ibrutinib concentrations had a ratio of 3.77, meaning that the actual exposure to anti-BTK drugs would have been underestimated by a factor 2.4 (i.e., 3.77/1.56) had only ibrutinib AUC been considered. Overall, 14 patients (16%) had ratios outside the 1.17–1.95 interval, implying a larger than +/−25% error on the actual exposure to anti-BTK drug. Finally, when the sampling strategies identified for ibrutinib AUC determination (T0–1–2–4, T0–1–4 and T0–2–4) were applied to estimate AUC_ANTI-BTK_ from both ibrutinib and DHD concentrations, good performances were obtained in development and validation datasets (Supplementary Table S1).

## 3. Discussion

There is a poor correlation between ibrutinib trough plasma concentration (C_min,ss_) and AUC_τ,ss_ (*r*^2^ = 0.25). Ibrutinib exposure for a specific patient cannot be estimated accurately from this unique concentration. This is due to the short half-life that also has a large inter-individual variability. The performances of the various schedules of blood sampling using the development dataset clearly show that an accurate determination of AUC_τ,ss_ requires at least three samples within the 0 (just before oral intake) and 4 h post-dose period. The T0–1–4 was associated with a slightly better prediction than the T0–2–4. The corresponding four-sample schedule (T0–1–2–4) gave an excellent estimation for all patients with 96% of patients within the +/−20% error interval. The prospective evaluation of these schedules on the validation cohort was consistent with the results found on the development dataset. The only difference was the inversion of the rank of T0–1–4 schedule with that of the T0–2–4. This may be due to the difference in ibrutinib dose between the two datasets. More likely, it is a statistical artefact due to a similar performance of both schedules. Overall, our analysis shows that a schedule based on three samples obtained at pre-dose, at 4 h, and an additional sample between 1–2 h, or better still, the 4-sample schedule including both 1 and 2 h, give a very accurate estimation for most patients with only 3/85 and 0/27 out of the +/−20% interval in the development and validation dataset, respectively. Although several four-sample strategies showed good performance, we decided to focus on T0–1–2–4 only, and not on strategies which include a T6 sample, such as T0–1–4–6. Indeed, the relevance of these strategies is penalized by the fact that it would require the patient to stay at the hospital for at least 6 h instead of four, for a similar quality of prediction of ibrutinib AUC.

Because ibrutinib half-life is short, we would not have anticipated the need for a T0 measurement. In order to facilitate TDM implementation for outpatients, the LSS without T0 were particularly evaluated, as these would enable patients to take ibrutinib at a regular fixed time at home, and be sampled during their time in hospital. However, the best sampling strategies included T0 (Figure 3) which requires that the patient take his/her ibrutinib treatment during his/her visit at the hospital. This highlights the fact that, even though the T0 concentration value is small and poorly correlated to exposure, it remains informative concerning the full PK profile of ibrutinib.

We also determined the concentrations of the main ibrutinib metabolite, dihydrodiol-ibrutinib, in every available sample. In our previous work [11], these data helped to determine the final structural PK model during model development. Indeed, when ibrutinib data alone were analyzed, ibrutinib oral bioavailability had to be fixed to a previously reported value (i.e., 3%). On the other hand, adding the DHD concentrations gave a good fit of all the data without fixing any parameter (apart from distribution volumes of DHD), as the model used metabolite formation to calculate the first-pass effect. Interestingly, in the present context of a maximum a posteriori Bayesian analysis, taking into account DHD plasma concentrations in three or four samples of the LSS did not improve the estimation of ibrutinib AUC. This might be explained by the high inter-individual variability of the parameters which determine the PK of DHD (64%, 64% and 50% for KA_DHD_, CL_MET_ and CL_DHD_ respectively), as well as the lack of correlation between residual errors. Indeed, these could make the PK of the two compounds too independent to be influenced by each other. In other words, whatever the PK profile of DHD, it does not tell us much about the PK of ibrutinib.

An estimate of the AUC of BTK inhibitor activity was obtained by combining the intrinsic activity of DHD (1/15 of that of ibrutinib), theoretical unbound fractions, and plasma concentrations of both compounds. Surprisingly, DHD carries an important anti-BTK activity, on average half that of ibrutinib. However, individual variations in this anti-BTK AUC are highly correlated to variations of ibrutinib AUC. Thus, considering AUC_ANTI-BTK_ instead of ibrutinib AUC is unlikely to refine PK/PD relationships, as they provide similar information concerning drug exposure. Nevertheless, if AUC_ANTI-BTK_ should appear as a better marker in future PK/PD studies, the same sampling strategy as for ibrutinib could be applied.

Since the drug’s registration, several analytical methods have been suggested to determine plasma ibrutinib concentrations [17,18,19,20,21]. However, the current work is the first to identify LSS facilitating whole plasma exposure determination and extending its implementation in routine clinical practice. The main limitation of this approach is the access to software able to obtain whole plasma exposure in a Bayesian framework. To date, NONMEM^®^ is the gold standard to perform these analyses, but we acknowledge that its usage is not optimal for a routine estimation of AUC and potential dose adaptation. The development of model-informed precision dosing tools and the inclusion of ibrutinib into software libraries is encouraged [22].

Thanks to the present work, it is now easier to interpret ibrutinib concentration data. Less sampling without compromising the quality of AUC estimation is good news both for the patient and for the biologist. To date, there was no validated method to obtain ibrutinib exposure, other than extensive sampling to determine the full PK profile of ibrutinib. This might have prevented the collection of data aiming to study relationships between ibrutinib exposure and toxicities in real-life settings, as compared to approaches where a single measure of trough concentration is a good surrogate for exposure (i.e., imatinib, pazopanib, sunitinib…). We think the present results will help to further explore PK/PD relationships in future studies, and promote the evaluation of ibrutinib TDM. Indeed, since toxicity is the most common reason for ibrutinib discontinuation [23], there is a need to develop strategies aiming to minimize ibrutinib exposure in clinical practice. Moreover, ibrutinib is sensitive to CYP3A inhibitors or inducers [24], thus current dosing strategies in cases of drug–drug interaction could be improved with a measure of ibrutinib exposure. Although routine implementation of TDM based on multiple time points and Bayesian analysis is heavier to implement than a simple measure of trough concentration, the example of immunosuppressant drugs proves the feasibility of this approach to individualize the dose of continuously taken oral drugs [25,26]. Thus, one could imagine that AUC-based TDM could be implemented for ibrutinib in order to manage drug exposure and avoid toxicities.

## 4. Materials and Methods

### 4.1. Patients

The limited sampling strategy was developed with the drug concentrations vs. time data from the patients of the PK-E3i study (No. NCT02824159). The aim of this study was to evaluate the association between toxicity and plasma concentrations of ibrutinib and idelalisib, as well as to develop a population pharmacokinetics model for ibrutinib and DHD. The details of the protocol have been published elsewhere [11].

### 4.2. Pharmacokinetic Data

PK exploration was made at steady state, one month after ibrutinib initiation. Patients were sampled in heparinized lithium tubes (5 mL) before (T0) and 30 min (T0.5), 1 h (T1), 2 h (T2), 4 h (T4) and 6 h (T6) after ibrutinib intake. Samples were immediately centrifuged (1400 g, room temperature, 10 min) and collected plasma was stored at −20 °C until analysis. Ibrutinib and DHD concentrations were determined using ultra-high performance liquid chromatography tandem mass spectrometry (UPLC-MS/MS, Waters, Saint-Quentin-en-Yvelines, France). The lower limit of quantification (LLOQ) was 0.98 ng/mL for both molecules. Concentrations under the LLOQ were set to LLOQ/2 (i.e., 0.49 ng/mL). Patients with less than six samples, missing DHD data, or outlier concentrations (i.e., that had been excluded from PK modelling [11] after graphical inspection) were excluded from the LSS analysis, as well as outlier patients with extreme values in C_min,ss_ (Grubbs’s test, *p* < 0.001).

### 4.3. Pharmacokinetic Model

A population PK model of ibrutinib and its main metabolite (DHD) was previously developed in our team on this cohort of 89 patients (PK-E3i study) [11]. Briefly, this model consists of a lagged zero-order infusion in a depot compartment, followed by a dual first-order absorption to the ibrutinib (KA_IBRU_) and DHD (KA_DHD_) central compartments. Both molecules distribute in a peripheral volume and are eliminated with first-order processes (CL_IBRU_/F and CL_DHD_/F). Additionally, the ibrutinib central compartment is joined to the DHD compartment with a metabolic clearance (CL_MET_/F). The distribution of parameters is lognormal, and residual errors on ibrutinib and DHD concentrations are proportional. A graphical representation of the model is reported in Supplementary Figure S1.

### 4.4. AUC Computation

The AUC (ng/mL·h) of ibrutinib (AUC_IBRU_) and DHD (AUC_DHD_) can be derived from the apparent parameter values using the following formulae:

(1) $${\mathrm{AUC}}_{\mathrm{IBRU}}=\;\frac{\mathrm{DOSE}\;}{{\mathrm{CL}}_{\mathrm{MET}}/F+{\;\mathrm{CL}}_{\mathrm{IBRU}}/F}\cdot \left(\frac{{\mathrm{KA}}_{\mathrm{IBRU}}}{{\mathrm{KA}}_{\mathrm{DHD}}+{\;\mathrm{KA}}_{\mathrm{IBRU}}}\right)$$

(2) $${\mathrm{AUC}}_{\mathrm{DHD}}=\;\frac{\mathrm{DOSE}\;}{{\mathrm{CL}}_{\mathrm{DHD}}/F}\cdot (\frac{{\mathrm{KA}}_{\mathrm{DHD}}}{{\mathrm{KA}}_{\mathrm{DHD}}+{\;\mathrm{KA}}_{\mathrm{IBRU}}}+\frac{{\mathrm{KA}}_{\mathrm{IBRU}}}{{\mathrm{KA}}_{\mathrm{DHD}}+{\;\mathrm{KA}}_{\mathrm{IBRU}}}\cdot \frac{{\mathrm{CL}}_{\mathrm{MET}}/F}{{\mathrm{CL}}_{\mathrm{MET}}/F+{\;\mathrm{CL}}_{\mathrm{IBRU}}/F}$$

Because little interest lies in the determination of AUC_DHD_ alone, a hybrid anti-BTK AUC was computed. It takes into account both AUC_IBRU_ and AUC_DHD_, as well as unbound fraction and intrinsic anti-BTK activity of the metabolite (1/15 of parent drug) according to the following formula:

(3) $${\mathrm{AUC}}_{\mathrm{ANTI}-\mathrm{BTK}}=(1-{\mathrm{fb}}_{\mathrm{IBRU}})\cdot {\mathrm{AUC}}_{\mathrm{IBRU}}+\frac{1}{15}\cdot \left(1-{\mathrm{fb}}_{\mathrm{DHD}}\right)\cdot {\mathrm{AUC}}_{\mathrm{DHD}}$$

The fraction of ibrutinib (fb_IBRU_) and DHD (fb_DHD_) bound to plasma protein was approximated to the previously estimated values of 97% and 91%, respectively [15].

### 4.5. Bayesian Analysis

Throughout the current study, “Bayesian analysis” refers to the analysis of concentration vs. time data using the maximum a posteriori Bayesian approach. The latter was conducted in the NONMEM^®^ software version 7.4.4 (ICON Development Solutions, Ellicott City, MD, USA) [27] with Pirana as a graphical interface and PsN toolkit [28]. Population PK parameters (i.e., THETA values, OMEGA and SIGMA matrices) of the model were fixed. Individual empirical Bayesian estimates were obtained with the First-Order Conditional Estimation + Interaction method and MAXEVAL = 0 plus $COVARIANCE UNCONDITIONAL options. Model code is given in Supplementary Table S2.

### 4.6. Actual AUC

Individual actual AUC for ibrutinib were obtained from the Bayesian analysis of the full PK profile (*n* = 6 samples). However, we investigated whether this analysis should take into account the concentration of ibrutinib alone or both ibrutinib and DHD concentrations. The choice of the best approach was made by comparing the goodness-of-fit (GOF) plots and the coefficient of determination (*r*^2^) between individual predicted and observed ibrutinib concentrations. The same strategy was applied to obtain actual AUC for DHD, by comparing the fit of DHD concentration data when both parent + metabolite or metabolite alone were taken into account. Finally, actual anti-BTK AUC was computed from actual ibrutinib AUC and actual DHD AUC.

### 4.7. Correlation between trough Ibrutinib Concentrations and Actual AUC

The Pearson’s coefficient of correlation between trough ibrutinib concentrations and the actual ibrutinib AUC was computed. Additionally, the linear regression equation was used to derive a model-independent AUC in order to compare its predictive performance of the actual AUC.

### 4.8. Limited Sampling Strategy

Datasets with every combination of sparse PK sampling from one to four samples were created. For each of these strategies, two Bayesian analyses were performed, considering concentrations of ibrutinib alone or ibrutinib + DHD. The estimated PK parameters for each of these analyses were derived into ibrutinib AUC. For each strategy, the performance of the estimation of ibrutinib AUC was evaluated by comparing the obtained AUC values (AUC_LSS_) to the actual AUC (AUC_actual_). Precision and bias were assessed by computation of root-mean-square error (RMSE) and mean percentage error (MPE), respectively.

(4) $$\mathrm{RMSE}\;=\sqrt{\frac{\sum {\left({\mathrm{AUC}}_{\mathrm{LSS}}-{\mathrm{AUC}}_{\mathrm{actual}}\right)}^{2}}{N}}\;$$

(5) $$\mathrm{MPE}=\frac{1}{N}\;\cdot \sum \left(\frac{{\mathrm{AUC}}_{\mathrm{LSS}}-{\mathrm{AUC}}_{\mathrm{actual}}}{{\mathrm{AUC}}_{\mathrm{actual}}}\right)$$

RMSE was normalized with the mean AUC_actual_ in order to be expressed as a percentage. Additionally, the percentage of patients with a residual error superior to +/−20% (P20) was computed.

### 4.9. Validation

The performance of the different sampling strategies was evaluated in an independent validation cohort. These concentration data were already published as the validation cohort of the PK model (*n* = 28 patients) [11]. The methods of sampling, concentration measurement and exclusion criteria were identical to those of the development cohort.

### 4.10. Anti-BTK AUC

In order to assess whether the actual AUC_ANTI-BTK_ and actual AUC_IBRU_ carry the same information concerning exposure to the anti-BTK drug, the distribution of the $\frac{{\mathrm{AUC}}_{\mathrm{ANTI}-\mathrm{BTK}}}{(1-{\mathrm{fb}}_{\mathrm{IBRU}})\cdot {\mathrm{AUC}}_{\mathrm{IBRU}}}$ ratio was also examined. More specifically, the percentage of patients outside of a +/−25% interval around the median value of the ratio was computed (as an arbitrary cut-off of what could possibly be a pharmacologically relevant difference). Finally, the LSS already obtained for the estimation of ibrutinib AUC was applied to ibrutinib + DHD concentration data in order to explore the predictive capacity of AUC_ANTI-BTK_ from a limited number of samples.

### 4.11. Software

Data management and post-hoc analyses were conducted in R version 4.0.3 (R Foundation for Statistical Computing, Vienna, Austria) with the tidyverse packages [29].

## 5. Conclusions

In the context of routine TDM of ibrutinib-treated patients, the best sampling strategy is T0–1–2–4 and an analysis of ibrutinib concentrations only. A sparser strategy removing the 1 h or 2 h point may still provide satisfactory results. Sampling strategies based on the sole measure of trough concentration or even two samples are precluded for an accurate prediction of ibrutinib AUC. Measuring DHD concentrations in order to refine the PK/PD relationship is unlikely to be relevant.

## Figures and Tables

**Table 1:** Patients’ characteristics in the development and validation cohort.

Characteristics		Development Cohort (*n* = 85)	Validation Cohort (*n* = 27)
		N (%)	
Disease	CLL	73 (86%)	23 (85%)
	MCL	10 (12%)	4 (15%)
	WM	2 (2%)	0 (0%)
Dose (mg) the day of PK exploration	140	1 (1%)	6 (22%)
	280	5 (6%)	7 (26%)
	420	70 (82%)	14 (52%)
	560	9 * (11%)	0 (0%)
Sex	Male	27 (31%)	11 (41%)
	Female	58 (68%)	16 (59%)
Prior treatment	No	19 (22%)	1 (4%)
	Yes	66 (78%)	26 (96%)
		Median (min–max)	
Age		69 (31–84)	68 (39–88)
Height		170 (148–187)	169 (150–186)
Weight		70 (40–106)	71 (48–91)
Number of day of treatment		32 (27–90)	203 (35–665)

CLL: chronic lymphocytic leukemia; MCL: mantle cell lymphoma; WM: Waldenström’s macroglobulinaemia; PK: pharmacokinetic; * One MCL patient initiated the treatment at 560 mg but received 420 mg the day of PK exploration.

**Table 2:** Performances of limited sampling strategies for ibrutinib AUC estimation, stratified by the number of concentration–time points and sorted by RMSE (first 6 entries) in the development cohort (*n* = 85).

Number of Points	Sampling Strategy	Mean Actual AUC (ng/mL·h)	RMSE (%)	MPE (%)	P20 (%)
1	T6	672	40.4%	+8.3%	59%
	T4	672	44.6%	+6.0%	81%
	T2	672	45.3%	−11.9%	62%
	T1	672	54.8%	+28.8%	72%
	T0.5	672	61.9%	+39.6%	71%
	T0 (LR) *	672	67.9%	+42.7%	71%
2	T1–4	672	22.8%	−7.2%	36%
	T2–4	672	25.8%	−6.0%	48%
	T1–6	672	30.0%	−9.1%	40%
	T2–6	672	30.0%	−12.0%	48%
	T0.5–4	672	31.6%	+4.2%	47%
	T0.5–6	672	34.2%	+5.1%	52%
3	T0–1–4	672	14.5%	+2.2%	25%
	T1–4–6	672	19.2%	−7.9%	31%
	T1–2–4	672	19.9%	−6.1%	28%
	T0.5–2–6	672	22.0%	−9.2%	28%
	T0–2–4	672	22.7%	−1.9%	29%
	T0.5–2–4	672	22.7%	−2.7%	31%
4	T0–1–2–4	672	11.0%	−0.3%	4%
	T0–1–4–6	672	13.1%	−0.5%	18%
	T0–0.5–2–4	672	13.3%	+3.1%	11%
	T0–0.5–1–4	672	15.3%	+4.1%	24%
	T1–2–4–6	672	15.7%	−8.2%	18%
	T0.5–2–4–6	672	17.1%	−4.5%	16%

MPE: mean percentage error; P20: proportion of patients with a residual error superior to +/−20%; RMSE: root-mean-square error; * AUC derived from the linear regression (LR) equation of actual ibrutinib AUC and trough concentration of ibrutinib.

## Data Availability

The data presented in this study are available on request from the corresponding author.

## Supplementary Materials

The following are available online at https://www.mdpi.com/1424-8247/14/2/162/s1, Table S1: Performance of several LSS to predict AUC_ANTI-BTK_. Figure S1: Diagram of the pharmacokinetic model. Table S2: NONMEM control stream of the PK model used for Bayesian estimation.
