# Supplementary material for: Limited Sampling Strategy for Determination of Ibrutinib Plasma Exposure: Joint Analyses with Metabolite Data
Source: Pharmaceuticals (Basel). 2021 Feb 18;14(2):162. doi: 10.3390/ph14020162 (PMC7922501; doi:10.3390/ph14020162)
Supplement: Supplementary file 1 [file pharmaceuticals-14-00162-s001.pdf]

pharmaceuticals

MDPI

SUPPLEMENTARY

## Supplementary Table S1: Performance of LSS to predict AUC ANTI-BTK

Development cohort

| N_point | LSS | N | MEAN | RMSE | MPE | nP20 | P20 |
|---|---|---|---|---|---|---|---|
| 4 | T0_1_2_4 | 85 | 31.2 | 10.3% | +0.8% | 2 | 2% |
| 3 | T0_1_4 | 85 | 31.2 | 11.3% | +0.3% | 6 | 7% |
| 3 | T0_2_4 | 85 | 31.2 | 16.2% | -1.1% | 14 | 16% |

Validation cohort

| N_point | LSS | N | MEAN | RMSE | MPE | nP20 | P20 |
|---|---|---|---|---|---|---|---|
| 4 | T0_1_2_4 | 27 | 27.2 | 5.9% | +2.8% | 0 | 0% |
| 3 | T0_2_4 | 27 | 27.2 | 7.3% | +2.9% | 1 | 4% |
| 3 | T0_1_4 | 27 | 27.2 | 15.8% | +0.5% | 5 | 19% |

## Supplementary Figure S1: Diagram of the pharmacokinetic model

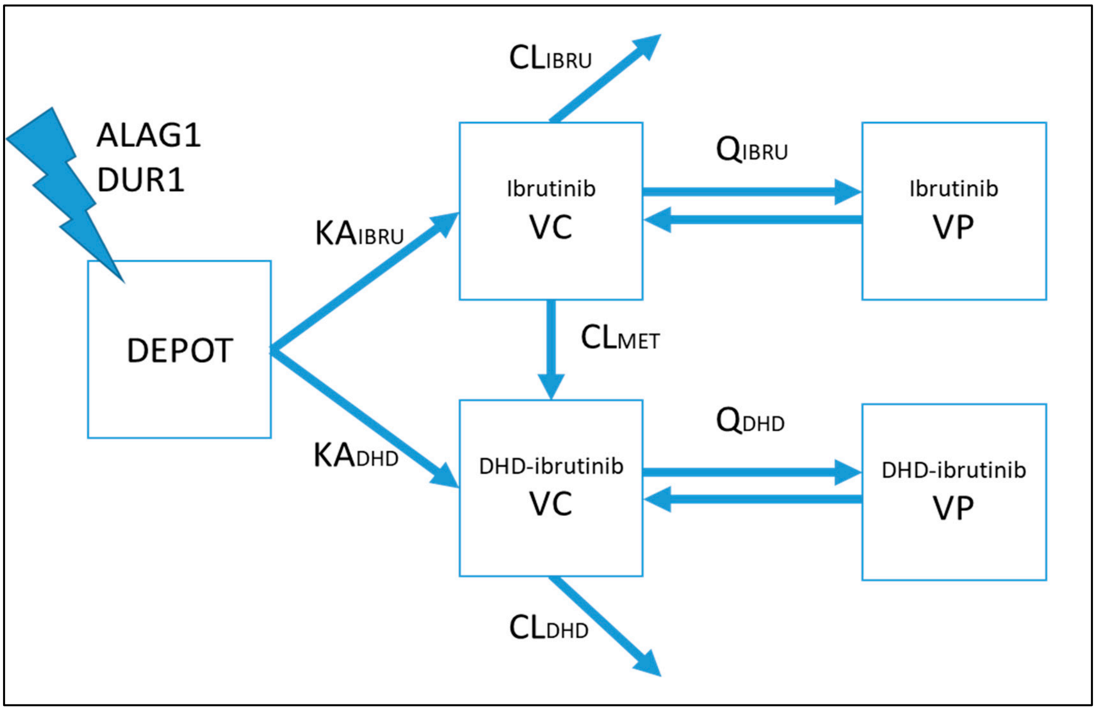


ALAG1, Lag time in compartment 1; DEPOT, Drug Depot; DHD, Dihydrodiol-ibrutinib; DUR1 Duration of infusion in compartment 1; KADHD, Absorption rate constant for DHD; KAIBRU, Absorption rate constant for ibrutinib; CLDHD, DHD clearance; CLIBRU, Ibrutinib clearance; CLMET, metabolic clearance; QDHD, intercompartmental DHD clearance, QIBRU intercompartmental ibrutinib clearance; VC; Central Volume of distribution; VP; Peripheral Volume of distribution.

Supplementary Table S2: Control stream PK model

```
$SUBROUTINES ADVAN5 SS5

$MODEL
COMP=(DEPOT,DEFDOSE)
COMP=(IBRU_CENT,DEFOBS)
COMP=(IBRU_PERIPH)
COMP=(DHDIBRU)
COMP=(DHDIBRU_PERIPH)

$PK
OCC1=0
OCC2=0
OCC3=0
OCC6=0
IF(MONTH.EQ.1) OCC1=1
IF(MONTH.EQ.2) OCC2=1
IF(MONTH.EQ.3) OCC3=1
IF(MONTH.EQ.6) OCC6=1

;IBRU
CLMET  = THETA(1) * EXP(ETA(6))
V2     = THETA(2) * EXP(ETA(2))
KA     = THETA(3)
Q      = THETA(4)
V3     = THETA(5) * EXP(ETA(1))
ALAG1  = THETA(6) * EXP(ETA(7))
D1     = THETA(7) * EXP(ETA(8))
CLIBRU = THETA(8) * EXP(ETA(4) + ETA(9)*OCC1 + ETA(10)*OCC2 + ETA(11)*OCC3 + ETA(12)*OCC6)

;DHD-IBRU
CLDHD = THETA(9) * EXP(ETA(3) + ETA(13)*OCC1 + ETA(14)*OCC2 + ETA(15)*OCC3 + ETA(16)*OCC6)
V4    = V2
V5    = V3
K14   = THETA(10) * EXP(ETA(5))
QDHD  = THETA(11)

K12 = KA
K23 = Q / V2
K32 = Q / V3
K24 = CLMET / V2
K40 = CLDHD / V4
K20 = CLIBRU / V2
K45 = QDHD / V4
K54 = QDHD / V5

S2 = V2 /1000
S4 = V4 /1000

$ERROR
```

```
DEL=0
IF (F.EQ.0) DEL=1
W=F+DEL
IF(CMT.EQ.2) Y=F+W*EPS(1)
IF(CMT.EQ.4) Y=F+W*EPS(2)
IPRED=F
IRES=DV-IPRED
IWRES=IRES/W

$THETA
149.622 FIXED ; 1 CL MET
1005.03 FIXED ; 2 V2
1.55664 FIXED ; 3 KA
171.094 FIXED ; 4 Q
1481.01 FIXED ; 5 V3
.238192 FIXED ; 6 ALAG1
.988671 FIXED ; 7 D1
241.899 FIXED ; 8 CLIBRU
180.972 FIXED ; 9 CLDHD
1.20812 FIXED ;10 K14
50.3012 FIXED ;11 QDHD

$OMEGA BLOCK(6)
0.465173 FIXED ; iiv V3
0.432131 0.512330000 ; iiv V2
0.220876 0.217668000 0.228809 ; iiv CLDHD
0.374779 0.421162000 0.195424 0.3658180  ; iiv CLIBRU
0.298891 0.287158000 0.156798 0.2487940 0.345464  ; iiv K14
0.000000 0.000591386 0.180014 0.0217671 0.129532 0.347017000 ; iiv CLMET

$OMEGA BLOCK(2)
0.501473 FIXED ; iiv ALAG1
0.282336 0.844851 ; iiv D1
$OMEGA BLOCK(1) 0.197216 FIXED;IOV CLIBRU
$OMEGA BLOCK(1) SAME ;IOV CLIBRU
$OMEGA BLOCK(1) SAME ;IOV CLIBRU
$OMEGA BLOCK(1) SAME ;IOV CLIBRU
$OMEGA BLOCK(1) 0.0637684 FIXED;IOV CLDHD
$OMEGA BLOCK(1) SAME ;IOV CLDHD
$OMEGA BLOCK(1) SAME ;IOV CLDHD
$OMEGA BLOCK(1) SAME ;IOV CLDHD

$SIGMA
0.128496 FIXED
0.0639887 FIXED

$ESTIMATION METHOD=1 INTERACTION MAXEVAL=0 SIGDIGITS=3 NOABORT

$COV UNCONDITIONAL
```
